# Trichiniasis in Germany

**Published:** 1864-05

**Authors:** 


### TRICHINIASIS IN GERMANY.

In the original department of this number will be found an interesting account, by a correspondent, of the recently discovered disease produced by the presence of Trichinæ in the human system. The *British Medical Journal* for Jan. 16th, 1864, contains some additional details, which we transfer to our pages, as we are confident they will interest our readers.

"A few months ago, there was a festive celebration at Hettstadt, a small country town near the Hartz Mountains, in Germany. Upwards of ore hundred persons sat down to an excellent dinner, and, having enjoyed themselves *more majorum*, separated, and went to their homes.

"Of these one hundred and three persons, mostly men in the prime of life, eighty-three are now in their graves; the majority of the twenty survivors linger with a fearful malady; and a few only walk apparently unscathed among the living, but in hourly fear of the outbreak of the disease which has carried away such numbers of their fellow-diners.

"They had all eaten of a poison at that festive board, the virulence of which far surpasses the reported effects of *aqua tophana*, or of the more tangible agents described in toxicological text-books. It was not a poison dug out of the earth, extracted from plants, or prepared in the laboratory of the chemist. It was not a poison administered by design or negligence. But it was a poison unknown to all concerned; and was eaten with the meat in which it was contained, and of which it formed a living constituent.

"When the festival at Hettstadt had been finally determined upon, and the dinner had been ordered at the hotel, the keeper of the tavern arranged his bill-of-fare. The introduction of the third course, it was settled, should consist, as usual in those parts of the country, of *Rostewurst und Gemuse*. The *Rostewurst* was, therefore, ordered at the butcher's the necessary number of days beforehand, in order to allow of its being properly smoked. The butcher, on his part, went expressly to a neighboring proprietor, and bought one of two pigs from the steward, who had been commissioned with the transaction by his master. It appears, however, that the steward, unfortunately, sold the pig which the master had not intended to sell, as he did not deem it sufficiently fat, or well-conditioned. Thus the wrong pig was sold, carried on a barrow to the butcher, killed and worked up into sausages. The sausages were duly smoked and delivered at the hotel. There they were fried and served to the guests at the dinner-table.

"On the day after the festival, several persons who had participated in the dinner were attacked with irritation of the intestines, loss of appetite, great prostration, and fever. The number of persons attacked rapidly increased; and great alarm was excited in the first instance by the apprehension of an impending epidemic of typhus fever or continued fever, with which the symptoms observed showed great similarity. But when, in some of the cases treated by the same physician, the features of the illness began to indicate at first acute peritonitis, then pneumonia of a circumscribed character, next paralysis of the intercostal muscles and the muscles in front of the neck, the hypothesis of septic fever, though sustained in other cases had to be abandoned with respect to these particular cases. Some unknown poison was now assumed to be at the bottom of the outbreak; and an active inquiry into all the circumstances of the dinner was instituted. Every article of food and material was subjected to a most rigid examination, without any result in the first instance. But when the symptoms in some of the cases invaded the muscles of the leg, particularly the calves of some of the sufferers, the description which Zenker had given of a case of fatal trichinous disease was remembered. The remnants of sausage, and of pork employed in its manufacture, were examined with the microscope and found to be literally swarming with encapsuled trichinæ. From the suffering muscles of several of the victims small pieces were excised, and under the microscope found charged with embryonic trichinæ in all stages of development. It could not be doubted any longer, that as many of the one hundred and three as had partaken of *Rostewurst* had been infested with trichinous disease by eating of trichinous pork, the parasites of which had, at least in part, escaped the effects of smoking and frying.

"This awful catastrophe awakened sympathy and fear throughout the whole of Germany. Most of the leading physicians were consulted in the interest of the sufferers, and some visited the neighborhood where most of the afflicted patients remained. But none could bring relief or cure. With an obstinacy unsurpassed by any other infectious or parasitic disease, trichiniasis carried its victims to the grave. Many anthelmintics were arrayed to destroy, if not the worms already in the flesh, at least those yet remaining in the intestinal canal. Picric acid was employed until its use seemed as dangerous as the disease; benzole, which had promised well in experiments upon animals, was tried, but was unavailing. As case after case died off, and the dissection of each proved the parasites to have been quite unaffected by the agents employed, the conviction was impressed upon every mind that a man afflicted with flesh-worm is doomed to die the slow death of exhaustion from nervous irritation, fever, and loss of muscular power, in systems essential to existence.

"But medical science had only just unravelled a mystery; and if it could not save the victims, it was determined at least to turn the occasion to the next best account. The cases were, therefore, observed with care, and chronicled with skill. All the multifarious features of the parasitic disease were registered in such a manner, that there can hereafter be no difficulty in the diagnosis of this disorder. A valuable diagnostic feature was repeatedly observed—namely, the appearance of the flesh-worm under the thin mucous membrane on the lower side of the tongue. The natural history of trichina in man was found to be the same as that in animals.

"All observations led to the conviction that the trichina encapsuled in the flesh is in the condition of puberty. Brought into the stomach, the calcareous capsule is digested with the flesh, and the trichina is set free. It probably feeds upon the walls of the intestines themselves; for the irritation of the intestines begins before the bringing forth of young trichinæ has taken place. Copulation is immediately effected; and within a few hours, or a short portion of days, from sixty to eighty live embryos leave the female, and begin their own career of destruction.

"This consists, in the first instance, in an attempt to pierce the walls of the intestinal canal. Great inflammation of the entire surface ensues, ending not rarely in death of the villous or mucous membrane, or in the formation of masses of pus on its surface. Sometimes there are bloody stools. But these severe symptoms only ensue when much trichinous meat has been eaten. When less has been consumed, pain and uneasiness in the abdomen are produced, accompanied, however, in all instances, by wasting fever and prostration. The embryos actually pierce the intestines, and are found free in the effusion, sometimes serous, sometimes purulent, which is always poured out into the abdominal cavity. Thence they again proceed towards the periphery of the body, pierce the peritoneum, causing great irritation, and sometimes peritonitis, to the extent of gluing the intestines together to a coherent mass. They next proceed to the muscles nearest to the abdomen; arrived at the elementary muscular fibres, which, under the microscope, appear as long cylinders with many transverse striæ, they pierce the membranes, enter the fibres, eat and destroy their striated contents, consume a great part of the granular detritus, moving up and down in the fibres until grown to the size necessary for passing into the quiescent state. They then roll up in spiral or other irregular windings, the bags of the muscular fibres collapse, and only where the trichinæ lie a calcareous mass is deposited, perhaps by the trichinæ themselves, which hardens into perfect capsules round the parasites. A muscular fibre may harbor one or several parasites; but every fibre invaded by a single parasite loses its character entirely, and becomes a bag of detritus from one end to the other.

"If it be remembered that one ounce of meat filled with trichinæ may form the stock from which, in a few days, three millions of worms may be bred; and that these worms will destroy in the course of a few weeks not less than two millions of striated muscular fibres—an idea of the extent of destruction produced by these parasites can be formed. We are not in a position to say to what proportion of the fifty or sixty pounds of muscle required for the performances of the human body these two millions of elementary fibres actually amount. In the muscles nearest to the abdomen, the destruction is sometimes so complete, that not a fibre free from parasites can be found. This amounts to complete paralysis. But death is not always produced by the paralysis; it is mostly the result of paralysis, peritonitis, and irritative fever combined. No case is known in which trichiniasis, after having declared itself, became arrested. All persons affected have either died, or are in such a state of prostration that their death is very probable.

"Most educated people in Germany have, in consequence of the Hettstadt tragedy, adopted the law of Moses, and avoid pork in any form. To some of the large pig-breeders in Westphalia, who keep as many as two thousand pigs, the sinking of the price of pork has been a ruinous—at the least, a serious—loss. In the dining-rooms of the hotels in the neighborhood of Hettstadt, notices are hung up announcing that pork will not be served in any form in these establishments. To counteract this panic, the farmer's club of the Hettstadt district gave a dinner at which no other meat but pork was eaten. But it has had no appreciable effect. The raw ham and sausages of Germany are doomed to extinction. The smoked and fried sausages must necessarily be avoided. * * * *

"In the South of Germany, some people now say that the Hungarian pigs are most frequently affected with trichinæ. This rumor, like the famous pork dinner of the farmer's club, may, however, have been set up with the intention of quieting apprehension about the native pigs. We have already mentioned the accident which befell the crew of a merchant vessel. They shipped a pig at Valparaiso, and killed it a few days before their arrival at Hamburg. Most of the sailors ate of the pork in one form or another. Several were affected with trichinæ and died. Of those whose fate could be inquired into, one only seems to have escaped the parasites. Another outbreak in Saxony has carried away twelve persons. A fourth wholesale poisoning by trichinæ is just reported from Offenbach, the Birmingham of Hesse-Darmstadt. Of upwards of twenty persons infected, three had already died when our correspondent's letter left. Numerous sporadic cases of fever, and epidemics of inscrutable peculiarity, but referred to an anomalous type of fever, are now claimed by medical authors, and with much show of reason, to have been outbreaks of trichiniasis, or flesh-worm disease. Several German physicians experimentalized with a view of finding a cure for this terrible disorder. Professor Eckhardt, at Giessen, we are told, has obtained permission to try the disease and supposed remedies upon a murderer under sentence of death. We have not been told whether his reward in case of success is to be a commutation of his capital sentence; but should hope this to be the case. The experiment, even if it should not have the romantic character indicated, will probably teach some curious details of the life of these parasites. Almost everywhere, the commonest rules of cleanliness are disregarded in the rearing of pigs. Yet pigs are naturally clean animals, avoiding, like dogs and cats, all contact with ordure. Though they burrow in the earth, and in summer wallow in the mud, they abhor the heaps of excrements mixed with straw in and upon which they are frequently kept. A due regard to cleanliness will prevent trichinæ in the pig. In wild boars, of which many are eaten in the country round the Hartz Mountains, trichina has never been found. Neither has it been met with in sheep, oxen, or horses. Beef is the safest of all descriptions of meat, as no parasites have ever been discovered in it. They have also never been found in the blood, brain, or heart, of those animals in whose striated muscles they love to reside."—*Am. Jour. of Med. Sciences.*

